# Retrospective analysis of antiretroviral therapy uptake and retention of male clients receiving methadone maintenance therapy in two provinces in Vietnam: potential synergy of the two therapies

**DOI:** 10.1186/s12954-017-0133-6

**Published:** 2017-02-17

**Authors:** Linh Thi Thuy Pham, Akiko Kitamura, Hoa Mai Do, Kim Anh Lai, Nhan Tuan Le, Van Thi Thuy Nguyen, Masaya Kato

**Affiliations:** 1 Health Management Training Institute, Hanoi University of Public Health, Hanoi, Vietnam; 2 Vietnam Country Office, World Health Organization, 304 Kim Ma, Hanoi, Vietnam; 3 Can Tho Provincial AIDS Center, Can Tho, Vietnam; 4 Hanoi Provincial AIDS Center, Hanoi, Vietnam

**Keywords:** Methadone maintenance therapy, Antiretroviral therapy, HIV/AIDS, People who inject drugs, Vietnam

## Abstract

**Background:**

Vietnam has a concentrated HIV epidemic with injection drug use being the dominant mode of HIV transmission. Vietnam has rapidly expanded antiretroviral therapy (ART) and methadone maintenance therapy (MMT). This study aims to analyze ART uptake and retention among male clients receiving MMT in Vietnam in the early phase of the MMT program.

**Methods:**

The male clients (age ≥18) who were newly enrolled in care or started ART at two HIV clinics in Hanoi (2009 to 2011) and three HIV clinics in Can Tho (2010 to 2012) were included for the analysis. The CD4 lymphocyte count at HIV care enrollment and ART initiation and retention on ART were retrospectively analyzed. The values of those receiving MMT were compared with the values of two groups: those in whom injection drug use (IDU) status was documented, but were not receiving MMT, and all male clients not receiving MMT. To analyze retention, survival analysis with log rank test and Cox proportional hazard model was used.

**Results:**

During the study period, 663 adult men were newly enrolled in HIV care (237 had IDU status documented) and 456 initiated ART (167 had IDU status documented). Among those who initiated ART, 28 were receiving MMT. At care enrolment, those receiving MMT had a median CD4 count of 230 (IQR 57–308) cells/mm^3^, while men self-reporting IDU and not receiving MMT and all men not receiving MMT had a median CD4 count of 158 (IQR 50–370) cells/mm^3^ and 143 (IQR 35–366) cells/mm^3^, respectively. At ART initiation, men receiving MMT had significantly higher CD4 count with median at 203 (IQR 64–290) cells/mm^3^ than men self-reporting IDU and not receiving MMT (80, IQR 40–220, cells/mm^3^, *p* = 0.038) and all men not receiving MMT (76, IQR 20–199, cells/mm^3^, *p* = 0.009). Those receiving MMT had a significantly higher retention rate than those self-reporting IDU but not receiving MMT (hazard ratio = 0.18, *p* = 0.019) and men not receiving MMT (hazard ratio = 0.20, *p* = 0.041).

**Conclusions:**

Our analysis suggests that men receiving MMT in Vietnam are achieving relatively early uptake and high retention rates on ART. The findings support potential benefits of integrating MMT and ART services in Vietnam.

## Background

Vietnam has a concentrated HIV epidemic with injection drug use (IDU) as the dominant mode of transmission [1, 2]. According to the HIV sentinel surveillance in 2013, HIV prevalence in people who inject drugs (PWID) was the highest (10.3%) among populations surveyed [1, 2]. Overall, HIV prevalence among PWID in Vietnam peaked in the early 2000s and gradually declined since, although the decline has been insignificant and the prevalence remains high in some provinces [1, 2]. According to national technical working group on Asian Epidemic Model, it is estimated that over 13,000 new HIV infection occur annually, and PWID is still projected to account for over 45% of new HIV infections in Vietnam towards 2020, if the interventions remain at the current level [2]. It was estimated that the number of PWID in 2009 was 271,000 [3]. Further intensified action is needed to reduce the risk of HIV transmission among this population.

In response, Vietnam has implemented a robust scale-up of harm reduction interventions, including needle and syringe program, opioid substitution therapy (OST), and condom promotion. However, the coverage of behavioral interventions has been modest—in 2013, needle and syringe program distributed only 98 needles and syringes per PWID and the rate of consistent condom use among PWID was only 41.2% [1]. To strengthen efforts for addressing the problem of injection drug use-associated HIV transmission, Vietnam piloted methadone maintenance therapy (MMT) in 2008, which resulted in successful outcomes [1, 2, 4]. MMT service was expanded to 80 sites by the end of 2013 resulting in the treatment of 15,542 clients in 30 provinces [1].

Since 2005, antiretroviral therapy (ART) was also rapidly expanded in Vietnam, and by the end of 2013, 82,687 people were receiving treatment [1, 2]. Despite these gains in treatment enrollment, late uptake of ART is common [1, 2, 5] and limits the full therapeutic and preventive benefits of ART. People living with HIV who inject drugs are likely to access ART with a lower CD4 count and with a higher prevalence of WHO stage 3 or 4 diseases, compared to those who do not inject drugs [5–7].

Previous studies from high-income countries have shown potential roles of MMT in facilitating uptake of, adherence to, and retention on ART among HIV-positive PWID [8–11]. However, to our knowledge, there have been a limited number of studies that examined HIV treatment parameters among people receiving MMT in resource-limited countries. The objective of our study was to retrospectively examine and document uptake of HIV care and treatment and retention on ART among male PWID who are receiving MMT in the early phase of MMT program in Vietnam. The study results are expected to contribute to optimizing service delivery of HIV and drug dependence treatment among PWID.

## Methods

### Study sites

The study was conducted in two outpatient clinics (Tu Liem District Health Center and Long Bien District Health Center) in Hanoi City, Northern Vietnam, and four outpatient clinics (Ninh Kieu District Health Center, Cai Rang District Health Center, O Mon District Health Center, and Can Tho Provincial Hospital) in Can Tho Province, Southern Vietnam. MMT-related data were obtained from MMT centers in the same districts in the two provinces. MMT and ART services are co-located in the same building at Tu Liem and Long Bien District Health Centers but were not co-located in the other districts studied. Can Tho Provincial Hospital was located in Ninh Kieu District.

### Study design

The following three indicators were retrospectively analyzed: (1) timeliness of HIV care enrollment as measured by CD4 count at new enrollment in the outpatient clinic, (2) timeliness of ART uptake as measured by CD4 count at ART initiation, and (3) retention on ART.

The values for the above indicators were compared between those receiving MMT and those not receiving MMT (the comparators). For the first two indicators (CD4 count at HIV care enrollment and CD4 count at ART initiation), an individual was considered “receiving MMT” if he was receiving MMT at the time of care enrollment and at ART initiation, respectively. For retention on ART, an individual was considered as having received MMT if he had received MMT at least 90 days while on ART (i.e., MMT treatment could be able to have potential effects on ART outcomes). For the comparison group (those not receiving/exposed to MMT), two types of comparators were used. The first was those whose drug use status was documented on the medical chart based on the self-report. The second comparator was all the males receiving ART at the study clinics. The rationale for using these two comparators was as follows: First, it is common for PWID in Vietnam not to disclose their IDU status to clinic staff due to stigma and discrimination associated with drug use and fear of being arrested and possibly taken to forced drug treatment centers [12–14]. Therefore, relying on IDU status documented on medical chart will likely underestimate the true PWID populations at the clinics. Second, it has been estimated that PWID account for a large majority (i.e., 60–70%) of males attending HIV outpatient clinics [3, 6], and thus, “all males” was considered a more sensitive but less specific inclusion criteria to provide proxy values as the comparator to those receiving MMT.

As there were very few females who inject drugs registered at the study sites, they were not included in the analysis.

### Inclusion and exclusion criteria

All adult males (age 18 or over) living with HIV who met the following time-range criteria were included in the analysis: (1) for the analysis of CD4 count at *care enrollment*, those who newly enrolled in study clinics from January 2009 to December 2011 in Hanoi and from June 2010 to December 2012 in Can Tho; (2) for the analysis of CD4 count at *ART initiation*, those who started ART from January 2009 to December 2011 in Hanoi and from June 2010 to December 2012 in Can Tho; and (3) those included in the analysis of CD4 count at ART initiation were also included in the analysis of *retention on ART*.

The data were collected in Can Tho one year later than in Hanoi to have meaningful duration of follow-up in order to analyze retention since MMT was introduced in Can Tho only in 2010 whereas MMT services were started in Hanoi in 2009. The actual time of data collection was in late 2012 in Hanoi and in January 2013 in Can Tho. Subjects who transferred in (from other HIV clinics while on ART) were excluded from the analysis of all three indicators; subjects who transferred out (to other HIV clinics while on ART) were excluded only from the analysis of retention.

### Data collection

The data were extracted from the patient charts by staff members of the Hanoi School of Public Health using data collection tools developed for this study. Data were entered twice by different staff members (double entries) to ensure the accuracy.

The following data were extracted at outpatient clinics: CD4 count at new enrollment for HIV care was defined as the first CD4 count measured within the 90 days before and 180 days after care enrollment; CD4 count at ART initiation was defined as the CD4 count measured within 180 days before and 30 days after the ART initiation date. In 2009, the national guideline on ART treatment defined CD4 threshold for ART initiation as 250 cells/mm^3^ [15]. Time frames for both MMT exposure and CD4 count were extensively consulted and agreed within the research team to reflect the service settings in the country. To analyze the retention on ART, the date of ART initiation and the end date of follow-up (i.e., censorship or attrition) were extracted from the patient’s chart. A patient was categorized as “attrition” when he or she died, was lost-to-follow-up (LTFU, defined as no clinic visit for 90 days from the last clinic visit by the Vietnam Ministry of Health based on WHO 2006 patient monitoring guidelines), or stopped ART (discontinuation of ART). Available socio-demographic parameters (i.e., age, sex, documented self-reported drug use history, residence, distance from residence to the clinic, means of HIV transmission) were also collected.

The following data were obtained from MMT service providers: the date MMT was initiated or re-initiated, and the date of discontinuation, if it occurred.

### Statistical analysis

Mann-Whitney tests were performed to compare CD4 counts among the MMT and non-MMT groups at care enrollment and at ART initiation. Survival analysis was conducted for ART retention using log rank test and Cox proportional hazard regression analysis and Kaplan-Meier curves was generated for illustration of ART retention of each group. The data were entered using Microsoft Excel, and statistical analysis was performed using STATA (version 12, StataCorp LP, Texas, USA).

### Ethics

The study protocol was approved by the Institutional Review Board of the Hanoi School of Public Health. The study was conducted with approval and cooperation of the Hanoi and Can Tho Provincial AIDS Centers.

## Results

### CD4 counts at HIV care enrollment

During the study period, 663 adult men were newly enrolled in the HIV outpatient clinics; 28 were receiving MMT (median age 34). Among the 635 men not receiving MMT (median age 34), 237 (37.3%) were self-reported injection drug users (median age 34). The men receiving MMT had median CD4 count of 230 (IQR 57–308) cells/mm^3^ at care enrollment, while those not receiving MMT with documented injection drug use had median CD4 count of 158 (IQR 50–370) cells/mm^3^. Among all men not receiving MMT, the median CD4 count was 143 (IQR 35–366) cells/mm^3^ (Table 1). Although those receiving MMT showed higher CD4 count values, the difference was not statistically significant. Three hundred six women not self-reported IDU were newly enrolled at care (median age 31) at the same time period with median CD4 count at 273 cells/mm^3^.

**Table 1:** CD4 count at HIV care enrollment among men receiving MMT vs two comparators not receiving MMT

	Number	Median age (IQR)	Median CD4 count at care enrollment (IQR), cells/mm^3^	*p* value
Males receiving MMT	28	34 (31–38)	230 (57–308)	0.541
Males whose injection drug use status was documented and not receiving MMT	237	34 (30–38)	158 (50–370)	
Males receiving MMT	28	34 (31–38)	230 (57–308)	0.308
Males not receiving MMT	633	34(29–37)	143 (35–366)	
Females (not self-reporting injection drug use)	306	31 (30–38)	273 (108–444)	

Females were not included in the analysis, since very few females reported injection drug use. Mann-Whitney test was used to compare the values between those receiving and not receiving MMT

### CD4 counts of participants at ART initiation

During the study period, 456 men initiated ART, among whom 28 were receiving MMT (median age 34). Among the 428 men not receiving MMT (median age 33), 167 had self-reported injection drug use (median age 32). Men receiving MMT had median CD4 count at 203 (IQR 64–290) cells/mm^3^ at ART initiation, which was significantly higher than the median CD4 count for men self-reporting injection drug use and not receiving MMT (80 cells/mm^3^, IQR 40–220, *p* = 0.038). The difference was even more pronounced between MMT and non-MMT users when self-reported IDU status was not considered (76 cells/mm^3^, IQR 20–199, *p* = 0.009). The majority of men had clinical stage 3 or 4 diseases (50 and 22%, respectively), and more than half (52.4%) had CD4 counts less than 100 cells/mm^3^ at ART initiation (Table 2). In this study, 265 women initiated ART whose median CD4 count at ART initiation was 184 cells/mm^3^ (IQR 74.5–274).

**Table 2:** CD4 count at ART initiation among men receiving MMT vs two comparators not receiving MMT

	Number	Median age (IQR)	Median CD4 count at ART initiation (IQR), cells/mm^3^	*p* value
Males receiving MMT	28	34 (31–38)	203 (57–273)	0.038
Males who self-reported injection drug use and not receiving MMT	167	32 (29–36)	80 (42–222)	
Males receiving MMT	28	34 (31–38)	203 (57–273)	0.009
Males not receiving MMT	428	33 (30–38)	76 (23–208)	
Females (not self-reporting injection drug use)	265	31 (28–36)	184 (68–278)	

Females were not included in the analysis, since very few females reported injection drug use. Mann-Whitney test was used to compare the values between those receiving and not receiving MMT

### Retention on ART

**Fig. 1:**
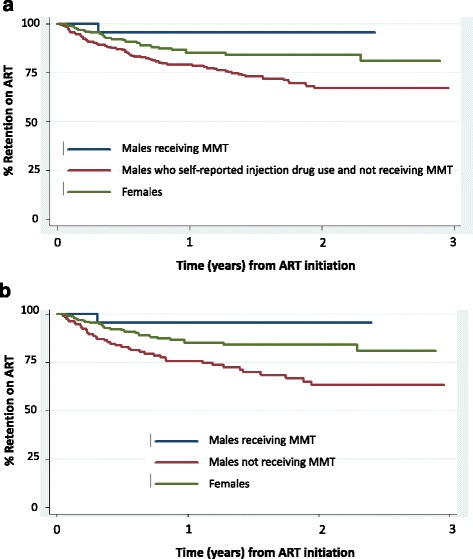
Kaplan-Meier curve of retention on ART among males receiving MMT. **a** Compared to males whose injection drug use was documented and not receiving MMT. **b** Compared to males not receiving MMT. Three hundred ninety-five men are included in the analysis, of which 28 were receiving MMT at ART initiation. There were 369 men who were not receiving MMT (**b**), among which 146 had injection drug use documented based on their self-report (**a**)

The retention on ART of 456 adult men was analyzed, 28 of whom were receiving MMT at least 90 days while on ART (median age 34). Among the 428 men not receiving MMT (median age 33), 167 had self-reported injection drug use documented in their charts (median age 32). Men receiving MMT had an attrition rate at 0.06 per person-year with only one case of attrition (stop treatment) over 25.4 person-years of follow-up; men self-reporting injection drug use but not receiving MMT had an attrition rate of 0.26 per person-year with 39 cases of attrition (24 deaths, 5 LTFU, and 10 stop treatment) over 207 person-years of follow-up; including all men regardless of self-reported IDU status, those not receiving MMT had an attrition rate of 0.20 per person-year with 88 cases of attrition (59 deaths, 6 LTFU, and 23 stopped treatment) over 483 person-years of follow-up. Kaplan-Meier survival curves for ART retention of each group are shown in Fig. 1. Cox proportional hazard regression analysis suggests men receiving MMT had significantly higher ART retention rates compared to men whose injection drug use was documented and not receiving MMT (hazard ratio = 0.18, LR*χ* ^2^ = 2,89, *p*=0.019, Fig. 1a) and also compared to all men not receiving MMT (hazard ratio = 0.20, LR*χ* ^2^ = 3.81, *p*=0.041, Fig. 1b).

## Discussion

The study showed that the men receiving MMT had earlier ART uptake (higher CD4 count at ART initiation) and higher retention on ART compared to those not receiving MMT in Hanoi and Can Tho. Since its introduction in 2008, the MMT program in Vietnam has produced positive outcomes and was therefore rapidly expanded [1, 2, 4]. However, to our knowledge, there are no previously published reports that examined the HIV care uptake and ART outcomes among those receiving MMT in Vietnam. While a number of literatures have reported synergistic effects of opioid substitution therapy and ART in high-income settings [8, 10, 11, 16–18], the present study contributes by providing evidence from Vietnam suggesting similar effects can also be expected in low- and middle-income settings.

It is important to note the limitations of the present study. First, our study analyzed the data retrospectively, and thus, causal relationship between MMT and ART parameters cannot be determined. It is also possible that those receiving or not receiving MMT may not be comparable. For example, it was suggested that commune police might have influenced the decision of some study subjects to access or not access MMT [19] resulting in selection bias since PWID must disclose their IDU status with the police to register for MMT. Second, the sample size of those receiving both ART and MMT was small (only 28). This is primarily because the study was conducted in the early phase of MMT program (in Vietnam, MMT was officially introduced in 2008), and thus, access to MMT was still limited among PWID. Moreover, only a small proportion (<30%) of PWID receiving MMT were HIV positive, and among these, only some were receiving ART. Third, the study was only conducted at six clinics in Hanoi and Can Tho, and thus, the findings cannot be generalized to other parts of the country. Finally, the study did not take into account other risk factors among the comparison groups such as sex work or male-to-male sex that could also influence the ART outcomes.

While recognizing these limitations, we believe that the following lines of evidence still support and suggest that MMT may play a role in facilitating the early uptake of HIV care and treatment and the ART retention. First, the PWID receiving MMT in our study had relatively high CD4 counts at ART initiation and high ART retention compared to national survey data collected during the same time period. For example, those receiving MMT in our study had median CD4 count of 219 cells/mm^3^ at ART initiation, while the survey covering 83 sites across 51 provinces in Vietnam reported that the median CD4 count at ART initiation was 97 cells/mm^3^ in 2011 and 105 cells/ mm^3^ in 2012 [5, 20]. The ART retention rate among the men receiving MMT in our study was 97%, while the national survey reported that the rate was 85% at 12 months post-ART initiation among adults who started ART in 2012 [1, 2]. Using females as the reference, we could see that regardless of IDU status, men not receiving MMT have the trend of ART retention lower than that of women. By taking MMT, the trend of ART retention of male PWID was reversed to even higher than that of females.

Two types of comparators were used in our analysis, with understanding that some PWID are not willing to self-report their injection drug use status, and thus, the status is not documented in medical chart. It would be ideal if men receiving MMT could be compared to the men who inject drugs but not receiving MMT; however, it is impossible to identify accurate status of injection drug use of individuals through a retrospective review of the medical chart. For this reason, men receiving MMT were compared to those whose injection drug use was documented on the medical charts based on their self-report. This method likely underestimates the true PWID population. Then, men receiving MMT were compared to all the males not receiving MMT. This approach likely includes some who do not inject drugs; however, available data [6, 7, 18] and estimation (unpublished data, national technical working on estimation and projection) suggest the majority of males receiving ART are PWID in Vietnam. The important point is that those receiving MMT had earlier ART uptake and higher ART retention than the comparator, regardless which comparator was used.

There have been other retrospective cohort studies assessing the effects of MMT similar in design to ours reporting the collection of data for a longer period [8, 16, 17]. Some of these studies, however, analyzed people receiving ART as a segment of overall MMT client populations [16, 17]. This approach might result in a larger sample size than ours; however, it would only be applicable in the settings where MMT had already been scaled up rather than in the early phase of MMT introduction as in our situation.

Overall, the findings of the present study are in line with those from previous studies and reviews, mostly from high-income settings, which reported exposure to MMT is positively associated with better ART uptake [8, 10, 11], ART adherence [8, 9, 16], and retention on ART [8, 17] among PWID populations. A recent review article also reported that concomitant administration of MMT and ART is associated with significant reduction in HIV-related and drug-related deaths [21]. It is plausible that regular exposure to opioid substitution services would create an effective opportunity to offer HIV testing on a periodic basis, to link those diagnosed as HIV positive to HIV care and treatment, and to provide support for ART adherence, all of which could potentially lead to earlier ART uptake and better treatment outcomes.

While more rigorously designed studies are needed to address the potential bias in our study and to examine the causal link between MMT exposure and ART outcomes, our results suggest that receiving both MMT and ART in Hanoi and Can Tho was associated with relatively high levels of ART uptake and retention. Together with existing lines of evidence, our findings support WHO’s recommendations to promote integrated service delivery of OST and ART for HIV-positive PWID [7].

## Conclusions

Our analysis suggests that the men receiving MMT in Vietnam are likely achieving relatively early uptake and high retention of ART, although our study was not designed to assess the causal link between MMT and ART. While positive synergy between OST and ART has been studied and documented extensively in high-income settings, our findings add new piece of evidence suggesting similar service delivery model is feasible and effective in the low- and middle-income settings. While further researches are needed to optimize the service delivery, it is recommended to further explore opportunities to promote synergy between OST and ART, possibly through integrating service delivery of HIV testing, ART, and MMT.

## Abbreviations

ART: Antiretroviral therapy
CD4: Cluster of differentiation 4 T lymphocytes
HIV: Human immunodeficiency virus
IDU: Injection drug use
LTFU: Lost-to-follow-up
MMT: Methadone maintenance therapy
OST: Opioid substitution therapy
PWID: People who inject drugs
WHO: World Health Organization
